# Preliminary Results of Iran Premature Coronary Artery Disease (IPAD study) and Implementation of IPAD Biobank: A Case-Control Study

**DOI:** 10.34172/aim.26603

**Published:** 2024-09-01

**Authors:** Ehsan Zarepur, Noushin Mohammadifard, Reza Nedaeinia, Masoumeh Sadeghi, Sedigheh Asgary, Mojgan Gharipour, Amirhossein Zarepur, Fateme Moslemi, Nizal Sarrafzadegan

**Affiliations:** ^1^Isfahan Cardiovascular Research Center, Cardiovascular Research Institute, Isfahan University of Medical Sciences, Isfahan, Iran; ^2^Hypertension Research Center, Cardiovascular Research Institute, Isfahan University of Medical Sciences, Isfahan, Iran; ^3^Cardiac Rehabilitation Research Center, Cardiovascular Research Institute, Isfahan University of Medical Sciences, Isfahan, Iran; ^4^Applied Physiology Research Center, Cardiovascular Research Institute, Isfahan University of Medical Sciences, Isfahan, Iran; ^5^Pediatric Cardiovascular Research Center, Cardiovascular Research Institute, Isfahan University of Medical Science, Isfahan, Iran; ^6^School of Medicine, Hormozgan University of Medical Sciences, Hormozgan, Iran; ^7^School of Medicine, Isfahan University of Medical Sciences, Isfahan, Iran; ^8^The Iranian Network of Cardiovascular Research

**Keywords:** Biological specimen banks, Coronary disease, Ethnic groups, Genetic research

## Abstract

**Background:**

Iran Premature Coronary Artery Disease (IPAD) is one of the first and largest studies of its kind in Asia that investigates different aspects of premature coronary artery disease (PCAD) in different ethnic groups in multiple cities. In this paper, we aim to describe the IPAD biobank establishment and present some preliminary results of the IPAD study.

**Methods:**

This case-control study was conducted on patients with documented angiography from different ethnicities in more than ten cities of Iran (males aged 60 years and below and females aged 70 years and below). Patients with coronary artery stenosis of more than 75% in at least one vessel (or left-main stenosis of more than 50%) were defined as the case group and patients with normal coronary angiography were considered as the control group. In addition to completing questionnaires and performing physical measurements, samples of serum, buffy coat, plasma, whole blood, saliva, urine, and feces were stored in the freezer at -80 °C.

**Results:**

The mean age of patients was 51.1±8.2, of which 43% were women. There were 1221 people in the control group and 1702 in the case group. Our enrollment is completed and data entry and transferring biosamples from different cities is still ongoing. About 30000 biosamples of different ethnicities are saved in the IPAD biobank.

**Conclusion:**

This study aims to develop a high-quality biobank and facilitate research on different aspects of PCAD, especially gene-environment interaction regarding ethnicity.

## Introduction

 Cardiovascular disease is one of the most important causes of death and disability in the world. The prevalence of premature coronary artery disease (PCAD) is increasing each year, which is related to lifestyle changes and genetic factors. The prevalence of this disease is also high in Iran, which is a reason for the importance of better understanding the factors affecting it. Studies have shown that cardiovascular risk factors do not have the same prevalence among different races and ethnicities.^1-4^ There are different ethnicities in Iran, including Fars (Persian), Azari (Azeri or Turk or Tork), Kurd (Kord), Arab, Lor (Lur), Gilak, Balouch (Balouchi or Balooch or Sistani), Turkman (Torkman or Turkmen), Qashqaei (Qashghai), and Bakhtiari. These ten ethnicities account for more than 95% of Iran’s population. In fact, due to differences in lifestyle, environmental, and genetic factors between across ethnicities, these factors can probably accelerate or slow the occurrence of cardiovascular events and thus affect the course of the disease.^1-4^

 Biobank is considered one of the most important tools for storing biospecimens, which are particularly prominent in medical research. Biobanks help to better understand a disease and its genetic and epigenetic factors.^5-9^ Different biobanks around the world are collecting biosamples. In addition to the establishment of national biobanks with hundreds of thousands of samples in developed countries, in recent years, the formation of biobanks on certain diseases has opened up new aspects of medicine. Biobanks of human biospecimens from patients with glioblastoma, congenital heart disease, patients with aortic aneurysms, or even patients with Lyme disease are examples of these.^10-14^

 Managing a biobank is a specialized job that requires extensive collaboration. Especially, considering the potential risks that working with a biobank can pose to employees, biobank formation is one of the most sensitive research efforts.^5-8^ Isfahan Cardiovascular Research Institute (ICRI), as one of the leading centers of forming biobanks in Iran with many years of experience in the long-term management of biobanks, established a biobank in accordance with standard protocols in this study. In this project, although samples were from different ethnicities and there was great difficulty in obtaining samples from different cities, it ultimately led to the formation of an invaluable biobank that can pave the way for many future researchers. It is one of the leading biobanks in the world regarding ethnicity and PCAD.

 Therefore, ICRI decided to collect and store high-quality biosamples from different ethnicities throughout Iran in the form of the Iran-Premature Coronary Artery Disease Biobank (IPAD study). The main aim of this biobank is to investigate the separate and combined effects of genetic and environmental factors on the risk of PCAD regarding ethnicity.

## Materials and Methods

###  Study Setting and Design

**Figure 1:**
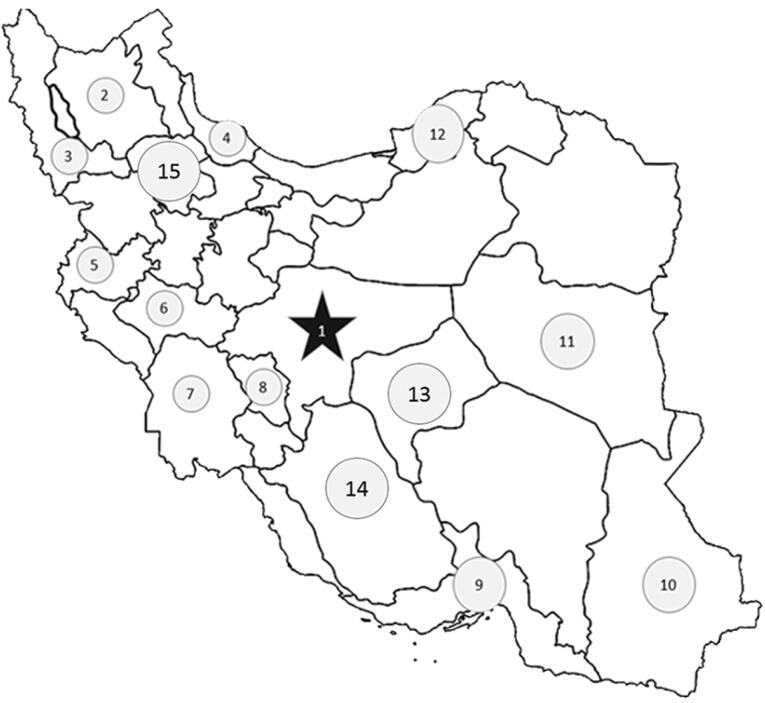
Different cardiac catheterization and coronary angiography units are included across the country. Cities: 1. Isfahan (Fars), 2. Tabriz and Maragheh (Azari), 3. Orumiyeh (Azari), 4. Rasht (Gilak), 5. Kermanshah (Kurd), 6. Khorramabad (Lor), 7. Ahvaz (Arab), 8. Shahrekord (Bakhtiari, Fars, Qashghaei), 9. Bandar Abbas (Fars), 10. Zahedan (Balouch), 11. Birjand (Fars), 12. Gorgan (Turkman), 13. Yazd (Fars), 14. Shiraz (Fars), 15. Zanjan (Azari). Orumiyeh, Bandar Abbas, and Gorgan will be included if it’s feasible/possible.

 IPAD is a multicenter case-control study that recruited patients from different cardiac catheterization and coronary angiography units in different cities to reach a sample size of 4000 in Iran. Individuals from different ethnicities such as Fars (Persian), Azari, Kurd, Arab, Lor, Bakhtiari, Balouch, Qashqaei, and Gilak were included in this study (males aged 60 years and below and females aged 70 years and below) who underwent coronary angiography (Figure 1). Also, the patient and his/her parents should be from the same ethnicity. Patients with a history of abnormal coronary angiography or coronary artery bypass graft (CABG) were excluded.

 Variables such as ethnicity, age, sex, socioeconomic characteristics, religion, physical activity, history of smoking, dietary habits, and family history were collected. The methodology of the IPAD study was previously reported.^15^

 Usually in every province of Iran, there is an educational hospital that has a cardiac angiography ward (cath-lab). Therefore, in this study, while communicating with these hospitals, necessary training was provided to conduct the study. Also, the main purpose of this study was to examine the data related to each ethnicity. Therefore, based on the percentage of each ethnicity in the country’s population, the number of selected ethnic groups was determined. In addition, for ethnicities with very small population, the number of samples in the study was determined in such a way that a proper analysis of the data can be done.

###  Inclusion and Exclusion Criteria

 The IPAD study included patients who underwent coronary angiography (males aged 60 years and below and females aged 70 years and below). Patients with at least 75% or more of a single coronary artery obstruction (or 50% or more of the left main coronary artery) were considered as the case group. Patients with normal arteries were in the control group. Patients with a registered history of coronary artery diseases such as balloon angioplasty, CABG, or percutaneous coronary intervention (PCI) were excluded from the study. During interim analysis, matching was evaluated (age, sex).

###  Quick Look

 Before completing questionnaires and performing physical measurements, the inclusion and exclusion criteria were re-checked and the date of service and name of the patient were recorded. A unique ID was assigned to each patient and necessary explanations were provided on obtaining blood, saliva, urine, and stool samples.

**Figure 2:**
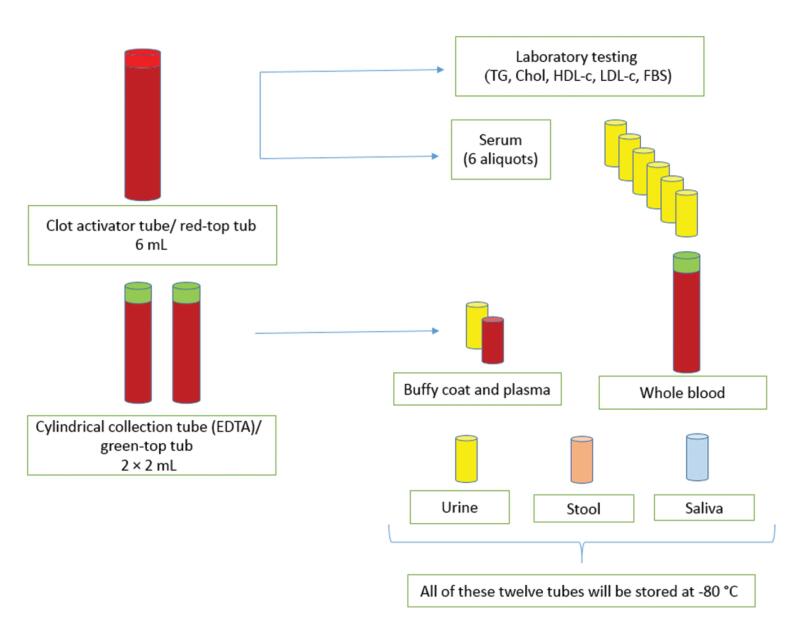
Processing and Storage of IPAD Biobank Biospecimens

 Required explanations of the procedure were given to the patient. The patient was in sitting position for at least five minutes prior to drawing the blood sample, and after sampling, was instructed to sit for five minutes to avoid any problems. Then, 10 mL of blood was taken according to standard protocols (4 mL in 2 × cylindrical collection tubes and 6 mL in 1 × clot activator tube) (Figure 2). Blood samples were evaluated for fasting blood sugar (FBS) and lipid profile and necessary processes were performed on biospecimens. All biological samples were transferred to the central lab of the ICRI and stored in freezers at -80 °C (Table 1).

**Table 1:** Description of Long-term Storage of Biospecimens Collected

**Tube**	**Description**
Cylindrical collection tube (EDTA/ green-top tube)	Separated into plasma and buffy coat.
Cylindrical collection tube (EDTA/ green-top tube)	2 mL, Stored at -80 °C
Urine	1.5 mL, Stored at -80 °C
Stool	Stored at -80 °C
Saliva	2 mL, Stored at -80 °C
Serum	Stored in 5 × 100 μL aliquots and 1 × 200 μL aliquot at -80 °C
Clot activator tube (red-top tube)	Serum separated from blood is used to measure fasting blood lipids profiles and FBS, and the rest of the serum is stored in 6 tubes. The tubes are stored at -80 °C.
Buffy coat with red blood cells combination	1 mL, stored at -80 °C
Plasma	1.5 mL, stored at -80°C

EDTA, Ethylenediaminetetraacetic acid; FBS, Fasting blood sugar.

###  Blood Pressure Measurement

 Blood pressure was measured on the right arm using a standard sphygmomanometer, in a sitting and comfortable position, after 30 minutes of rest. The person should not have smoked or climbed the stairs half an hour before the blood pressure measurement. The person sat comfortably and kept his right hand at the level of the heart. An arm cuff was used to measure blood pressure. The sphygmomanometer was tied on the person’s arm in such a way that it was a few centimeters higher than the cubital area. The sphygmomanometer cuff was suitable for the patient and not too small or too large for the patient’s arm.

###  Labeling Tubes

 The labeling of tubes was intended to be simple and understandable. Packages including different tubes were delivered to the executive team in each city (Figure 2). All labels had the IPAD abbreviation (left, top), the code of sample taken from a patient (right, top), and the patient’s unique barcode ID (bottom, middle). The patient’s barcode ID was a 5-digit number, the first digit being assigned to different cities and the next digit being the patient code.

###  Sample Collection

####  Cylindrical Collection Tube (EDTA)/ Green-Top Tub

 These two tubes were filled with 2 cc of whole blood and then the tube was shaken for about 10 minutes. The first tube was centrifuged as soon as possible. The separated plasma was then poured into a 1.5 cc tube and the buffy coat was transferred to a different 1.5 cc tube. The other tube was filled with 2 cc of whole blood and then the tube was shaken for about 10 minutes. These three tubes (whole blood, plasma, and buffy coat) were stored in their own cryoboxes in the freezer at -80 °C.

####  Urine

 Each package contained a sterile collection container. At first, the sterile collection container was filled by the patient and then the sample was transferred from the sterile collection container to the smaller freezer container for storage at -80 °C.

####  Stool

 Using a spoon in the stool collection container, a small sample from three different sites of stool was transferred to a smaller container. The stool should not touch the inside of the toilet or be mixed with water, soap, toilet paper, and napkins.

 If the patient had diarrhea or had been on antibiotics in the past month, the sample was not collected or stored.

####  Saliva

 The patient was in fasting condition. The sample was poured into a 1.5-mL container, the lid was closed tightly and transferred to the freezer (-80 °C) as soon as possible. If this was not possible, the sample was kept in a cold environment such as a refrigerator (-4 °C) and then transferred to the freezer (-80 °C).

####  Clot Activator Tube/ Red-Top Tub

 This tube was filled with 6 mL of whole blood. The blood was allowed to clot for 45 minutes at room temperature. After this, the tube was centrifuged (15 minutes at 3000 g). Serum was separated from the clot as quickly as possible, and 150 µL of the serum was sent to the laboratory for FBS, triglyceride, cholesterol, LDL-c, and HDL-c tests. The remaining serum was transferred to 5 × 100 µL and 1 × 200 μL aliquot tubes and stored at -80 °C. The clot activator tube was discarded after separating the serum. The serum was separated from the clot as quickly as possible since not separating the serum from the clot leads to changes in some of the factors in the serum.

###  Preparation

 For measuring lipid profile and FBS, the patients were in fasting condition for about 12 hours. It was also noted that drinking a small amount of water was not prohibited. Prolonged fasting itself may lead to potential changes in FBS and lipid profile. Therefore, the blood sample in this study was not taken under fasting conditions for more than 16 hours.

 Blood samples were drawn carefully to ensure that the red blood cells were not damaged (red blood cell lysis). It was important to ensure that the blood sample does not contaminate the sampler so that other patient samples may not be contaminated or affected during operation. If the sampling room was far from the laboratory or freezers, full compliance with cold chain was considered by the executive teams. If the serum sample was not sufficiently separated after centrifugation, the serum sample was separated and the clot activator tube was centrifuged again.

###  Freezers

 In this study, the principles of working with freezers were followed. Proper temperature control of the freezers was one of the most important considerations for the executive team. If the temperature drops and the samples and freeze-thaw cycles melt, it will undoubtedly affect the quality of many of the samples, and in some cases will cause substantial changes in some of the biomarkers.

 In this study, all samples of a patient were not kept in only one freezer. Indeed, one patient’s biospecimens were stored in at least two different freezers. Specimens were stored separately in their own cryoboxes in the freezers. Each cryobox was specific to one type of sample.

 Periodic monitoring of freezers and recording their temperature changes were among the most important principles of working with freezers. The freezers were cleaned every six months and turned off and on again annually. An emergency power system was provided for the freezers. All biological samples were transferred from different cities to the central lab of the ICRI periodically and full compliance with the cold chain was maintained.

## Results

 The mean age of patients whose samples are stored in the IPAD biobank is 51.1 ± 8.2 (49 ± 3 for men and 54 ± 2 for women), of which 43% are women. So far, there are 1221 people in the control group and 1702 in the case group. So far (September 2021), About 30 000 samples of different ethnicities are saved in the biobank (Table 2).

**Table 2:** Description of Long-term Storage of Biospecimens Collected

**Tube**	**Number of Samples in IPAD Biobank**
Cylindrical collection tube (EDTA/ green-top tube)	≈ 2800
Buffy coat with red blood cells combination	≈ 2800
Plasma	≈ 2800
Urine	≈ 2400
Stool	≈ 2100
Saliva	≈ 2200
Serum	≈ 17000

EDTA, Ethylenediaminetetraacetic acid.

 Patients with single-vessel disease accounted for 46.2% of patients in the case group and the result of the angiographic report of 313 (26.7%) was two-vessel disease (Table 3). So far, the largest number of samples has been of Fars ethnicity (1441 patients). Recruiting is still ongoing in Tabriz (Azari), Maragheh (Azari), Zanjan (Azari), Rasht (Gilak), Kermanshah (Kurd), Khorramabad (Lor), Ahvaz (Arab), Zahedan (Balouch), Yazd (Fars), and Shiraz (Fars).

**Table 3:** Report of Angiography of the Study Population

**Ethnicities**	**Normal** **n (%)**	**Premature CAD (1168)**		
		**SVD ** **n (%)**	**2VD ** **n (%)**	**3VD ** **n (%)**
Fars	559 (38.8)	406 (28.2)	204 (14.2)	206 (14.3)
Azari	3 (37.5)	2 (25.0)	1 (12.5)	2 (25.0)
Kurd	114 (42.1)	45 (16.6)	48 (17.7)	61 (22.5)
Lor	20 (51.3)	7 (17.9)	5 (12.8)	5 (12.8)
Bakhtiari	64 (36.0)	49 (27.5)	34 (19.1)	20 (11.2)
Qashqaei	52 (44.1)	25 (21.2)	19 (16.1)	18 (15.3)
Gilak	1 (33.3)	1 (33.3)	1 (33.3)	0 (0.0)
Arab	3 (30.0)	5 (50.0)	1 (10.0)	1 (10.0)
Turkman	0 (0.0)	0 (0.0)	0 (0.0)	0 (0.0)
Balouch	1 (33.3)	0 (0.0)	0 (0.0)	2 (66.7)
Total	817	540	313	315

SVD, single-vessel disease; 2VD, two-vessel disease; 3VD, three-vessel disease. Note: Calculated sample for each ethnicity is as follows (proportionally according to the distribution of each ethnic group): 2000 Fars patients, 510 Azaries, 400 Kurds, 250 Gilaks, 140 Arabs, 140 Lors, 140 Turkmans, 140 Bakhtiaries, 140 Qashghaeis, and 140 Balouches.

## Discussion

 Here, we described the design and implementation (sampling, processing, and storage) of the IPAD study biobank. In the present study, the diversity of each patient’s biosamples and saving all of them are among IPAD strengths. In fact, saving stool, saliva, urine, and blood samples at the same time is not common in different studies and is difficult.^11-14^ Blood samples in this study were separated into plasma, buffy coat, and serum. This type of saving allows us to study many factors and avoid the freeze-thaw cycle. This study is a multicenter project that despite the difficulty in implementing, will provide valuable data.

 There are different ethnicities living in Iran with different lifestyles and so far no comprehensive study has been conducted to investigate the different risk factors across these ethnicities.^15^ This study investigates the risk factors of PCRD across different ethnicities in more than ten cities of Iran. Although there have been limited studies in the past, none have comprehensively covered most Iranian ethnicities or established a biobank.^16-18^

 Being multi-centric is one of the prominent strengths of the present study. In the present study, patients from different ethnicities and cities are recruited. Data and biosamples are obtained at the patients’ place of residence. Researchers in the IPAD study could recruit different patients of different ethnicities by visiting large hospitals in major cities. However, because of differences in the environment, lifestyle, and many different risk factors,^1-4^ theresearchers decided to go to different cities and implement the study in each city.

 Also, considering the dominance of the Fars ethnicity in the country (between 50% and 70% of the population),^15^ Fars people from four different cities (Isfahan, Birjand, Yazd, Shahrekord) were included in this study to investigate possible differences and gain a better understanding of gene-environment interaction.^19^ These cities are located in different parts of Iran with probably different risk factors and different environments.^16-19^

 Indeed, this study only includes PCAD, and no other cardiac or non-cardiac diseases like some other small population-based biobanks. Therefore, even among ethnic groups whose sample size is relatively small compared to the total population of the study, we will be able to analyze various fac0tors. Although the sample size is small for some ethnicities, patients are selected exactly according to the study criteria. In many biobanks, this cannot be implemented. For example, in establishing the Adult Congenital Heart Disease Biobank (ACHD),^12^ with some of its results published in 2016, Opotowsky et al discussed how they established the ACHD biobank and collected the blood and urine samples of patients with congenital heart disease. From the beginning of the study in 2012 until the publication of the article, 11 000 samples were collected from 650 individuals aged 18 to 80 years and stored in the ACHD biobank in various containers and tubes. ACHD includes many diseases and heterogeneity can lead to difficulty in interpreting the results in the ACHD biobank.

 Also, in the ACHD biobank,^12^ blood and urine samples were collected from each patient and after performing some laboratory tests, the rest of the samples were stored in a biobank (plasma, buffy coat, serum, and urine). One of the limitations of the ACHD biobank and some other biobanks is the low diversity of samples. However, in the present study, different samples are stored and the diversity of each patient’s biosamples is one of the IPAD biobank strengths. Stool, saliva, urine, and blood samples are not collected simultaneously in many biobanks,^11-14^ especially the stool specimen, which is more difficult to collect than other specimens. So, researchers in this study will be able to analyze different samples and better understand PCAD regarding ethnicity.

 Blood samples in this study are separated into plasma, buffy coat, serum, and whole blood and stored in the IPAD biobank for years. This type of saving the samples is common and some other biobanks are saving these samples.^5-8,11-14^ The presence of a buffy coat, which has a high leukocyte count in a smaller volume of blood, greatly improves the quality of genetic testing while occupying less space in the biobank. On the other hand, having six aliquots of each person’s serum in different tubes allows the researchers to prevent freeze-thaw cycles and the test results will be more accurate.

 Another advantage of this study is keeping the samples at a temperature of -80 °C. Indeed, most samples can be kept for a few days at -4 °C and a couple of months at -20 °C. But in this study, the specimens will be kept for years with high quality and can be used in various research projects. This is important in future studies because new designed tests and methods in the future can be used and compared with the old ones very precisely using biobank samples. Also, the ICRI, as one of the oldest and leading centers in Iran in biobank formation and long-term maintenance, has a good experience and it can guarantee the quality of samples. In addition, combined genetic, laboratory investigations, and data from the questionnaires can greatly help to understand PCAD.

## Limitations and Suggestions

 This study has limitations like any other study. Storing the samples in liquid nitrogen can preserve the samples for longer years and therefore, not keeping the samples in liquid nitrogen is one of the limitations of this study. In addition, due to some limitations, especially the long-time storage of samples in the formation of each biobank and its costs, some samples are stored in just one tube which can affect the quality of the sample over different freeze-thaw cycles.

 The authors suggest that smaller ethnicities with a small percentage of the population of Iran should also be considered. The ethnicities studied in this project account for more than 90% of the population of Iran. Due to the limitations, it was not possible to examine smaller ethnicities such as Georgians, Kourmanj, Taleshi, etc. However, even if there were plans to include these ethnicities in the study, there might not have been enough patients to enroll. Also, fecal sample collection in this study was difficult and in some cases impossible. Therefore, the number of fecal samples is smaller than the other samples.

## Conclusion

 Establishment of a biobank is one of the most important and difficult steps in medical research. This paper can help other researchers in establishing biobanks. The IPAD biobank is one of the largest biobanks in the study of ethnicity and PCAD in the world. It is also one of the highest quality and largest biobanks in the Middle East region. It has the potential to be a good source of research for many years.
